# Supplementary material for: Pyruvate Dehydrogenase Kinase 1 inhibition mediated oxidative phosphorylation enhancement in cartilage promotes osteoarthritis progression
Source: BMC Musculoskelet Disord. 2023 Jul 20;24:597. doi: 10.1186/s12891-023-06585-6 (PMC10357736; doi:10.1186/s12891-023-06585-6)
Supplement: Supplementary file 1 — Additional file 1. [file 12891_2023_6585_MOESM1_ESM.zip › BP.Gsea.1653623667859/GOBP_SPROUTING_ANGIOGENESIS.html]

Details for gene set GOBP\_SPROUTING\_ANGIOGENESIS[GSEA]

|  || Dataset | 55457\_collapsed\_to\_symbols.cls.cls #OA\_versus\_normal.cls.cls #OA\_versus\_normal\_repos |
| Phenotype | cls.cls#OA\_versus\_normal\_repos |
| Upregulated in class | normal |
| GeneSet | GOBP\_SPROUTING\_ANGIOGENESIS |
| Enrichment Score (ES) | -0.4464865 |
| Normalized Enrichment Score (NES) | -1.9689485 |
| Nominal p-value | 0.0 |
| FDR q-value | 0.039201874 |
| FWER p-Value | 0.4 |
Table: GSEA Results Summary

  

Fig 1: Enrichment plot: GOBP\_SPROUTING\_ANGIOGENESIS      
 Profile of the Running ES Score & Positions of GeneSet Members on the Rank Ordered List

  

| SYMBOL | TITLE | RANK IN GENE LIST | RANK METRIC SCORE | RUNNING ES | CORE ENRICHMENT || 1 | CIB1 | calcium and integrin binding 1 [Source:HGNC Symbol;Acc:HGNC:16920] | 436 | 0.691 | -0.0161 | No |
| 2 | ITGB1BP1 | integrin subunit beta 1 binding protein 1 [Source:HGNC Symbol;Acc:HGNC:23927] | 631 | 0.611 | -0.0153 | No |
| 3 | FGF18 | fibroblast growth factor 18 [Source:HGNC Symbol;Acc:HGNC:3674] | 969 | 0.525 | -0.0280 | No |
| 4 | AKT1 | AKT serine/threonine kinase 1 [Source:HGNC Symbol;Acc:HGNC:391] | 1192 | 0.478 | -0.0329 | No |
| 5 | GREM1 | "gremlin 1, DAN family BMP antagonist [Source:HGNC Symbol;Acc:HGNC:2001]" | 1255 | 0.467 | -0.0255 | No |
| 6 | MAP2K5 | mitogen-activated protein kinase kinase 5 [Source:HGNC Symbol;Acc:HGNC:6845] | 1391 | 0.444 | -0.0245 | No |
| 7 | ANGPT1 | angiopoietin 1 [Source:HGNC Symbol;Acc:HGNC:484] | 1467 | 0.433 | -0.0191 | No |
| 8 | PIK3CB | "phosphatidylinositol-4,5-bisphosphate 3-kinase catalytic subunit beta [Source:HGNC Symbol;Acc:HGNC:8976]" | 2186 | 0.340 | -0.0663 | No |
| 9 | SRPX2 | sushi repeat containing protein X-linked 2 [Source:HGNC Symbol;Acc:HGNC:30668] | 2208 | 0.337 | -0.0592 | No |
| 10 | VEGFC | vascular endothelial growth factor C [Source:HGNC Symbol;Acc:HGNC:12682] | 2239 | 0.335 | -0.0528 | No |
| 11 | RECK | reversion inducing cysteine rich protein with kazal motifs [Source:HGNC Symbol;Acc:HGNC:11345] | 2307 | 0.326 | -0.0495 | No |
| 12 | SEMA5A | semaphorin 5A [Source:HGNC Symbol;Acc:HGNC:10736] | 3308 | 0.220 | -0.1219 | No |
| 13 | PARVA | parvin alpha [Source:HGNC Symbol;Acc:HGNC:14652] | 3499 | 0.202 | -0.1315 | No |
| 14 | MIA3 | MIA SH3 domain ER export factor 3 [Source:HGNC Symbol;Acc:HGNC:24008] | 3550 | 0.198 | -0.1303 | No |
| 15 | NRP1 | neuropilin 1 [Source:HGNC Symbol;Acc:HGNC:8004] | 3704 | 0.185 | -0.1374 | No |
| 16 | ROBO1 | roundabout guidance receptor 1 [Source:HGNC Symbol;Acc:HGNC:10249] | 3737 | 0.182 | -0.1352 | No |
| 17 | ALOX5 | arachidonate 5-lipoxygenase [Source:HGNC Symbol;Acc:HGNC:435] | 3776 | 0.179 | -0.1335 | No |
| 18 | PDCD10 | programmed cell death 10 [Source:HGNC Symbol;Acc:HGNC:8761] | 3896 | 0.165 | -0.1384 | No |
| 19 | PKM | pyruvate kinase M1/2 [Source:HGNC Symbol;Acc:HGNC:9021] | 3963 | 0.159 | -0.1395 | No |
| 20 | NGFR | nerve growth factor receptor [Source:HGNC Symbol;Acc:HGNC:7809] | 4156 | 0.142 | -0.1508 | No |
| 21 | HDAC5 | histone deacetylase 5 [Source:HGNC Symbol;Acc:HGNC:14068] | 4336 | 0.125 | -0.1615 | No |
| 22 | FGFBP1 | fibroblast growth factor binding protein 1 [Source:HGNC Symbol;Acc:HGNC:19695] | 4409 | 0.120 | -0.1640 | No |
| 23 | FGF1 | fibroblast growth factor 1 [Source:HGNC Symbol;Acc:HGNC:3665] | 4436 | 0.118 | -0.1629 | No |
| 24 | SEMA3E | semaphorin 3E [Source:HGNC Symbol;Acc:HGNC:10727] | 4815 | 0.086 | -0.1902 | No |
| 25 | FGF16 | fibroblast growth factor 16 [Source:HGNC Symbol;Acc:HGNC:3672] | 5013 | 0.072 | -0.2038 | No |
| 26 | GPLD1 | glycosylphosphatidylinositol specific phospholipase D1 [Source:HGNC Symbol;Acc:HGNC:4459] | 5103 | 0.064 | -0.2091 | No |
| 27 | MEOX2 | mesenchyme homeobox 2 [Source:HGNC Symbol;Acc:HGNC:7014] | 5128 | 0.062 | -0.2093 | No |
| 28 | HDAC9 | histone deacetylase 9 [Source:HGNC Symbol;Acc:HGNC:14065] | 5183 | 0.058 | -0.2120 | No |
| 29 | E2F8 | E2F transcription factor 8 [Source:HGNC Symbol;Acc:HGNC:24727] | 5527 | 0.031 | -0.2381 | No |
| 30 | ESM1 | endothelial cell specific molecule 1 [Source:HGNC Symbol;Acc:HGNC:3466] | 5692 | 0.017 | -0.2504 | No |
| 31 | IL12B | interleukin 12B [Source:HGNC Symbol;Acc:HGNC:5970] | 5991 | -0.004 | -0.2736 | No |
| 32 | AGTR1 | angiotensin II receptor type 1 [Source:HGNC Symbol;Acc:HGNC:336] | 6122 | -0.014 | -0.2834 | No |
| 33 | PPP1R16B | protein phosphatase 1 regulatory subunit 16B [Source:HGNC Symbol;Acc:HGNC:15850] | 6283 | -0.026 | -0.2953 | No |
| 34 | SYNJ2BP | synaptojanin 2 binding protein [Source:HGNC Symbol;Acc:HGNC:18955] | 6471 | -0.040 | -0.3088 | No |
| 35 | TBXA2R | thromboxane A2 receptor [Source:HGNC Symbol;Acc:HGNC:11608] | 6651 | -0.053 | -0.3215 | No |
| 36 | IL12A | interleukin 12A [Source:HGNC Symbol;Acc:HGNC:5969] | 6930 | -0.074 | -0.3412 | No |
| 37 | CEACAM1 | CEA cell adhesion molecule 1 [Source:HGNC Symbol;Acc:HGNC:1814] | 6960 | -0.077 | -0.3415 | No |
| 38 | JAK1 | Janus kinase 1 [Source:HGNC Symbol;Acc:HGNC:6190] | 6995 | -0.079 | -0.3421 | No |
| 39 | FGF2 | fibroblast growth factor 2 [Source:HGNC Symbol;Acc:HGNC:3676] | 7183 | -0.095 | -0.3542 | No |
| 40 | ENG | endoglin [Source:HGNC Symbol;Acc:HGNC:3349] | 7213 | -0.097 | -0.3540 | No |
| 41 | RAMP2 | receptor activity modifying protein 2 [Source:HGNC Symbol;Acc:HGNC:9844] | 7247 | -0.099 | -0.3540 | No |
| 42 | CREB3L1 | cAMP responsive element binding protein 3 like 1 [Source:HGNC Symbol;Acc:HGNC:18856] | 7324 | -0.104 | -0.3572 | No |
| 43 | LEF1 | lymphoid enhancer binding factor 1 [Source:HGNC Symbol;Acc:HGNC:6551] | 7467 | -0.116 | -0.3653 | No |
| 44 | ADGRA2 | adhesion G protein-coupled receptor A2 [Source:HGNC Symbol;Acc:HGNC:17849] | 7588 | -0.126 | -0.3714 | No |
| 45 | NR2E1 | nuclear receptor subfamily 2 group E member 1 [Source:HGNC Symbol;Acc:HGNC:7973] | 7660 | -0.132 | -0.3735 | No |
| 46 | LOXL2 | lysyl oxidase like 2 [Source:HGNC Symbol;Acc:HGNC:6666] | 7675 | -0.133 | -0.3711 | No |
| 47 | BMP4 | bone morphogenetic protein 4 [Source:HGNC Symbol;Acc:HGNC:1071] | 7816 | -0.142 | -0.3784 | No |
| 48 | CARD10 | caspase recruitment domain family member 10 [Source:HGNC Symbol;Acc:HGNC:16422] | 7971 | -0.153 | -0.3864 | No |
| 49 | APLNR | apelin receptor [Source:HGNC Symbol;Acc:HGNC:339] | 8001 | -0.155 | -0.3846 | No |
| 50 | AKT3 | AKT serine/threonine kinase 3 [Source:HGNC Symbol;Acc:HGNC:393] | 8008 | -0.156 | -0.3810 | No |
| 51 | E2F2 | E2F transcription factor 2 [Source:HGNC Symbol;Acc:HGNC:3114] | 8090 | -0.162 | -0.3831 | No |
| 52 | MMRN2 | multimerin 2 [Source:HGNC Symbol;Acc:HGNC:19888] | 8140 | -0.166 | -0.3826 | No |
| 53 | S100A1 | S100 calcium binding protein A1 [Source:HGNC Symbol;Acc:HGNC:10486] | 8304 | -0.178 | -0.3907 | No |
| 54 | SLIT2 | slit guidance ligand 2 [Source:HGNC Symbol;Acc:HGNC:11086] | 8449 | -0.190 | -0.3970 | No |
| 55 | GHSR | growth hormone secretagogue receptor [Source:HGNC Symbol;Acc:HGNC:4267] | 8472 | -0.191 | -0.3938 | No |
| 56 | TJP1 | tight junction protein 1 [Source:HGNC Symbol;Acc:HGNC:11827] | 8523 | -0.195 | -0.3926 | No |
| 57 | RHOA | ras homolog family member A [Source:HGNC Symbol;Acc:HGNC:667] | 8601 | -0.201 | -0.3934 | No |
| 58 | TEK | TEK receptor tyrosine kinase [Source:HGNC Symbol;Acc:HGNC:11724] | 8623 | -0.203 | -0.3897 | No |
| 59 | VEGFB | vascular endothelial growth factor B [Source:HGNC Symbol;Acc:HGNC:12681] | 8673 | -0.206 | -0.3882 | No |
| 60 | PIK3R3 | phosphoinositide-3-kinase regulatory subunit 3 [Source:HGNC Symbol;Acc:HGNC:8981] | 8801 | -0.217 | -0.3924 | No |
| 61 | IL10 | interleukin 10 [Source:HGNC Symbol;Acc:HGNC:5962] | 8855 | -0.221 | -0.3908 | No |
| 62 | JCAD | junctional cadherin 5 associated [Source:HGNC Symbol;Acc:HGNC:29283] | 8989 | -0.231 | -0.3952 | No |
| 63 | ABL1 | "ABL proto-oncogene 1, non-receptor tyrosine kinase [Source:HGNC Symbol;Acc:HGNC:76]" | 9081 | -0.238 | -0.3961 | No |
| 64 | PIK3C2A | phosphatidylinositol-4-phosphate 3-kinase catalytic subunit type 2 alpha [Source:HGNC Symbol;Acc:HGNC:8971] | 9301 | -0.256 | -0.4065 | No |
| 65 | PLK2 | polo like kinase 2 [Source:HGNC Symbol;Acc:HGNC:19699] | 9482 | -0.273 | -0.4135 | No |
| 66 | SMAD1 | SMAD family member 1 [Source:HGNC Symbol;Acc:HGNC:6767] | 9905 | -0.312 | -0.4383 | Yes |
| 67 | ADTRP | androgen dependent TFPI regulating protein [Source:HGNC Symbol;Acc:HGNC:21214] | 9987 | -0.320 | -0.4363 | Yes |
| 68 | NAXE | NAD(P)HX epimerase [Source:HGNC Symbol;Acc:HGNC:18453] | 9994 | -0.320 | -0.4284 | Yes |
| 69 | EPHB4 | EPH receptor B4 [Source:HGNC Symbol;Acc:HGNC:3395] | 10097 | -0.330 | -0.4278 | Yes |
| 70 | MAP3K3 | mitogen-activated protein kinase kinase kinase 3 [Source:HGNC Symbol;Acc:HGNC:6855] | 10121 | -0.333 | -0.4209 | Yes |
| 71 | STARD13 | StAR related lipid transfer domain containing 13 [Source:HGNC Symbol;Acc:HGNC:19164] | 10183 | -0.339 | -0.4168 | Yes |
| 72 | EPHA2 | EPH receptor A2 [Source:HGNC Symbol;Acc:HGNC:3386] | 10219 | -0.343 | -0.4106 | Yes |
| 73 | EFNB2 | ephrin B2 [Source:HGNC Symbol;Acc:HGNC:3227] | 10298 | -0.350 | -0.4076 | Yes |
| 74 | VSTM4 | V-set and transmembrane domain containing 4 [Source:HGNC Symbol;Acc:HGNC:26470] | 10444 | -0.366 | -0.4094 | Yes |
| 75 | EPN2 | epsin 2 [Source:HGNC Symbol;Acc:HGNC:18639] | 10446 | -0.367 | -0.3999 | Yes |
| 76 | TNN | tenascin N [Source:HGNC Symbol;Acc:HGNC:22942] | 10713 | -0.395 | -0.4104 | Yes |
| 77 | ANXA1 | annexin A1 [Source:HGNC Symbol;Acc:HGNC:533] | 10824 | -0.407 | -0.4084 | Yes |
| 78 | GATA2 | GATA binding protein 2 [Source:HGNC Symbol;Acc:HGNC:4171] | 11058 | -0.439 | -0.4151 | Yes |
| 79 | FUT1 | fucosyltransferase 1 (H blood group) [Source:HGNC Symbol;Acc:HGNC:4012] | 11086 | -0.442 | -0.4057 | Yes |
| 80 | VEGFD | vascular endothelial growth factor D [Source:HGNC Symbol;Acc:HGNC:3708] | 11095 | -0.443 | -0.3948 | Yes |
| 81 | SEMA6A | semaphorin 6A [Source:HGNC Symbol;Acc:HGNC:10738] | 11126 | -0.446 | -0.3855 | Yes |
| 82 | EGR3 | early growth response 3 [Source:HGNC Symbol;Acc:HGNC:3240] | 11207 | -0.458 | -0.3798 | Yes |
| 83 | KLF2 | Kruppel like factor 2 [Source:HGNC Symbol;Acc:HGNC:6347] | 11350 | -0.478 | -0.3784 | Yes |
| 84 | KDR | kinase insert domain receptor [Source:HGNC Symbol;Acc:HGNC:6307] | 11454 | -0.496 | -0.3735 | Yes |
| 85 | THBS1 | thrombospondin 1 [Source:HGNC Symbol;Acc:HGNC:11785] | 11498 | -0.501 | -0.3638 | Yes |
| 86 | FBXW7 | F-box and WD repeat domain containing 7 [Source:HGNC Symbol;Acc:HGNC:16712] | 11524 | -0.505 | -0.3526 | Yes |
| 87 | TDGF1 | teratocarcinoma-derived growth factor 1 [Source:HGNC Symbol;Acc:HGNC:11701] | 11531 | -0.505 | -0.3399 | Yes |
| 88 | PTK2B | protein tyrosine kinase 2 beta [Source:HGNC Symbol;Acc:HGNC:9612] | 11618 | -0.520 | -0.3331 | Yes |
| 89 | NR4A1 | nuclear receptor subfamily 4 group A member 1 [Source:HGNC Symbol;Acc:HGNC:7980] | 11709 | -0.535 | -0.3262 | Yes |
| 90 | PDPK1 | 3-phosphoinositide dependent protein kinase 1 [Source:HGNC Symbol;Acc:HGNC:8816] | 11839 | -0.557 | -0.3217 | Yes |
| 91 | CEMIP2 | cell migration inducing hyaluronidase 2 [Source:HGNC Symbol;Acc:HGNC:11869] | 11971 | -0.588 | -0.3166 | Yes |
| 92 | FOXC2 | forkhead box C2 [Source:HGNC Symbol;Acc:HGNC:3801] | 12218 | -0.650 | -0.3189 | Yes |
| 93 | ACVRL1 | activin A receptor like type 1 [Source:HGNC Symbol;Acc:HGNC:175] | 12242 | -0.656 | -0.3036 | Yes |
| 94 | FLT4 | fms related receptor tyrosine kinase 4 [Source:HGNC Symbol;Acc:HGNC:3767] | 12396 | -0.705 | -0.2972 | Yes |
| 95 | HMOX1 | heme oxygenase 1 [Source:HGNC Symbol;Acc:HGNC:5013] | 12421 | -0.714 | -0.2804 | Yes |
| 96 | ADAMTS9 | ADAM metallopeptidase with thrombospondin type 1 motif 9 [Source:HGNC Symbol;Acc:HGNC:13202] | 12428 | -0.717 | -0.2622 | Yes |
| 97 | ITGA5 | integrin subunit alpha 5 [Source:HGNC Symbol;Acc:HGNC:6141] | 12474 | -0.737 | -0.2465 | Yes |
| 98 | GLUL | glutamate-ammonia ligase [Source:HGNC Symbol;Acc:HGNC:4341] | 12527 | -0.762 | -0.2307 | Yes |
| 99 | SRF | serum response factor [Source:HGNC Symbol;Acc:HGNC:11291] | 12549 | -0.773 | -0.2122 | Yes |
| 100 | NOTCH1 | notch receptor 1 [Source:HGNC Symbol;Acc:HGNC:7881] | 12555 | -0.776 | -0.1923 | Yes |
| 101 | ITGB1 | integrin subunit beta 1 [Source:HGNC Symbol;Acc:HGNC:6153] | 12566 | -0.780 | -0.1728 | Yes |
| 102 | VEGFA | vascular endothelial growth factor A [Source:HGNC Symbol;Acc:HGNC:12680] | 12629 | -0.813 | -0.1564 | Yes |
| 103 | PTGS2 | prostaglandin-endoperoxide synthase 2 [Source:HGNC Symbol;Acc:HGNC:9605] | 12641 | -0.820 | -0.1359 | Yes |
| 104 | JMJD6 | "jumonji domain containing 6, arginine demethylase and lysine hydroxylase [Source:HGNC Symbol;Acc:HGNC:19355]" | 12658 | -0.832 | -0.1154 | Yes |
| 105 | CDC42 | cell division cycle 42 [Source:HGNC Symbol;Acc:HGNC:1736] | 12681 | -0.852 | -0.0949 | Yes |
| 106 | EPN1 | epsin 1 [Source:HGNC Symbol;Acc:HGNC:21604] | 12833 | -1.037 | -0.0797 | Yes |
| 107 | KLF4 | Kruppel like factor 4 [Source:HGNC Symbol;Acc:HGNC:6348] | 12848 | -1.058 | -0.0532 | Yes |
| 108 | PGF | placental growth factor [Source:HGNC Symbol;Acc:HGNC:8893] | 12850 | -1.059 | -0.0257 | Yes |
| 109 | HDAC7 | histone deacetylase 7 [Source:HGNC Symbol;Acc:HGNC:14067] | 12870 | -1.131 | 0.0023 | Yes |
Table: GSEA details [plain text format]

  

Fig 2: GOBP\_SPROUTING\_ANGIOGENESIS      
 Blue-Pink O' Gram in the Space of the Analyzed GeneSet

  

Fig 3: GOBP\_SPROUTING\_ANGIOGENESIS: Random ES distribution      
 Gene set null distribution of ES for **GOBP\_SPROUTING\_ANGIOGENESIS**
